# Supplementary material for: Impact of Iodine Electrodeposition on Nanoporous Carbon Electrode Determined by EQCM, XPS and In Situ Raman Spectroscopy
Source: Nanomaterials (Basel). 2023 May 4;13(9):1545. doi: 10.3390/nano13091545 (PMC10180143; doi:10.3390/nano13091545)
Supplement: Supplementary file 1 [file nanomaterials-13-01545-s001.zip › nanomaterials-2343051-supplementary.pdf]

nanomaterials

Supporting Information

Impact of Iodine Electrodeposition on Nanoporous Carbon Electrode Determined by EQCM, XPS and In Situ Raman Spectroscopy

Equations for EQCM calculations

$$\Delta f_{cal} = \frac{\Delta Q * C_f * M}{F * z_i} \quad (1)$$

$$M = \frac{M_{sodium} - M_{iodide}}{2} \quad (2)$$

Additional tables and figures regarding the G- and D-band during stable cycling

Table S1. Fit parameters of the fits in **Figure 3.** The equation for the fit is. $y = k \cdot x$

Band parameter	k	Δk (2 σ confidence)
ω_G	-0.07 [cm^{-1}]	± 0.01 [cm^{-1}]
ω_D	0.73 [cm^{-1}]	± 0.02 [cm^{-1}]
Γ_G	-0.06 [cm^{-1}]	± 0.01 [cm^{-1}]
Γ_D	-1.25 [cm^{-1}]	± 0.02 [cm^{-1}]
I_D/I_G	-0.0115	± 0.0002

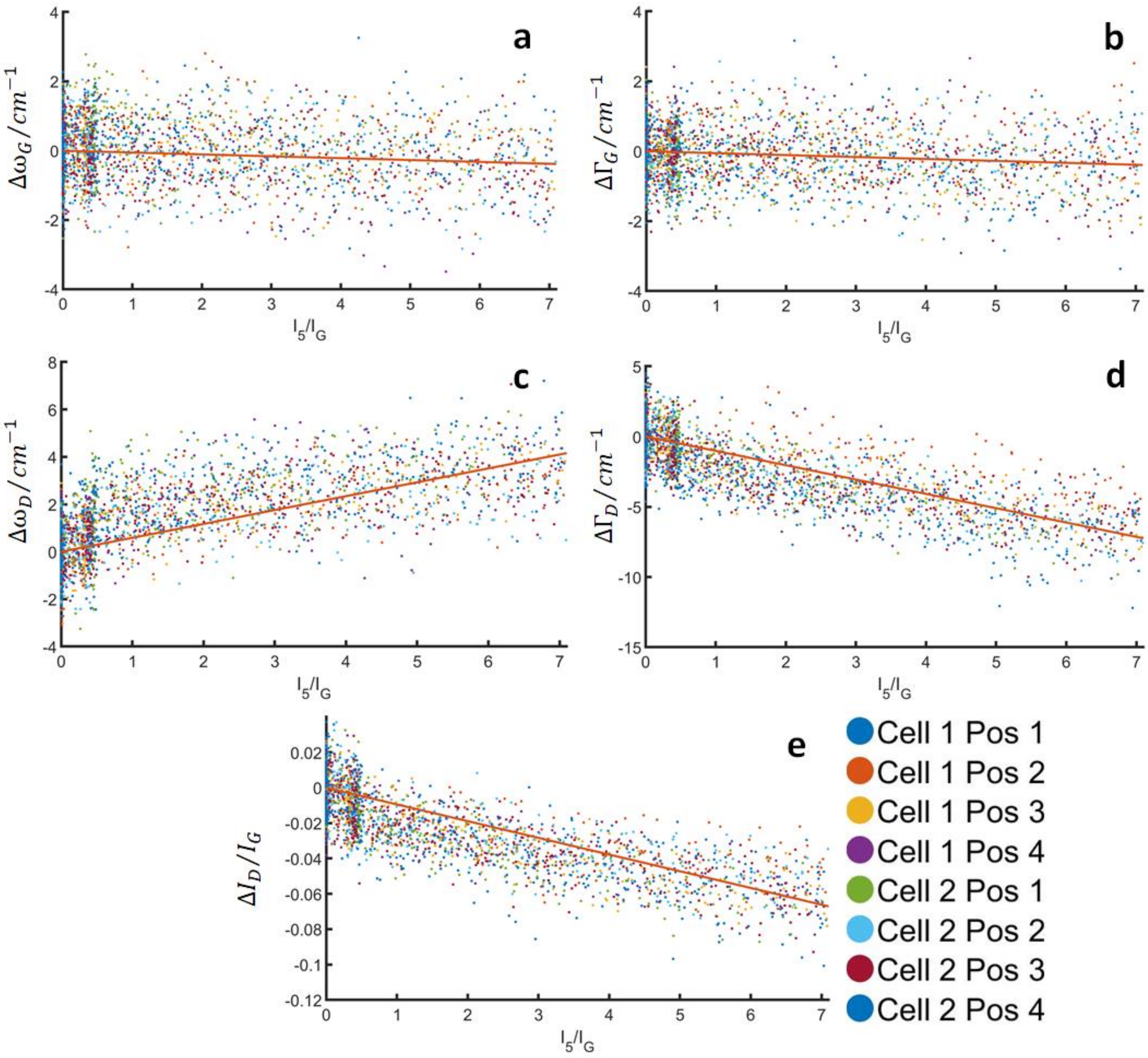

Figure S1. Changes to the G- and D-band parameters as polyiodides are generated on the surface. The intensity ratio of the main I_5 band (160 cm^{-1}) and the G-band (1600 cm^{-1}) are used as a measure for the amount of polyiodides formed. (a) G-band position, (b) G-band width, (c) D-band position, (d) D-band width (e) intensity ratio of the D-/G-band.

Table S2. Fit parameters of the fits in Figure S 1. The equation for the fit is $y = k \cdot x$

Band parameter	k	Δk (2 σ confidence)
ω_G	-0.05 [cm^{-1}]	± 0.01 [cm^{-1}]
ω_D	0.59 [cm^{-1}]	± 0.01 [cm^{-1}]
Γ_G	-0.06 [cm^{-1}]	± 0.01 [cm^{-1}]
Γ_D	-1.02 [cm^{-1}]	± 0.02 [cm^{-1}]
I_D/I_G	-0.0115	± 0.0002

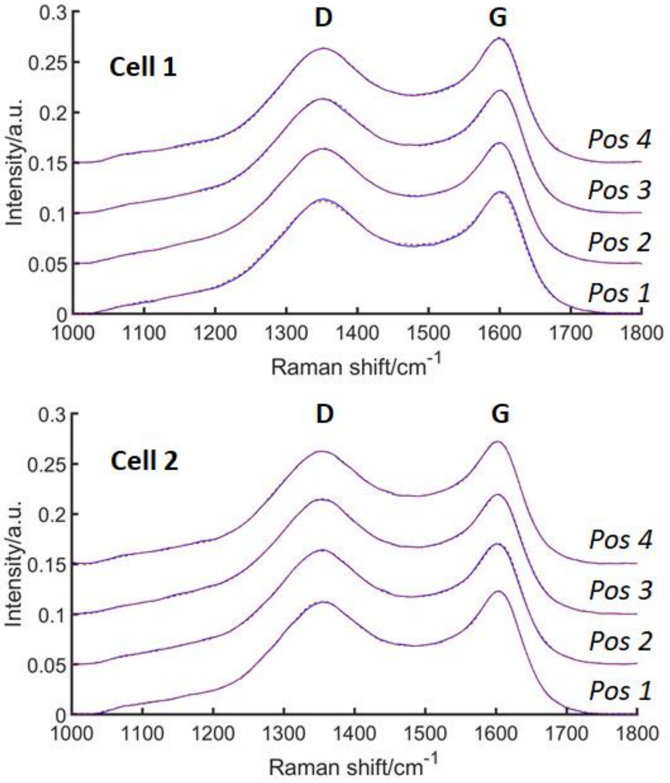

Figure S2. Averaged Raman spectra between -0.075 to +0.075 V at the beginning (blue line) and end (red, broken line) of a cycle form both cells and all measurement positions shown in Figure 3.

Table S3. G- and D-band parameter changes after one cycle calculated form the spectra in Figure S2.

Cell/Position	$\Delta\omega_G$/cm^{-1}	$\Delta\omega_D$/cm^{-1}	$\Delta\Gamma_G$/cm^{-1}	$\Delta\Gamma_D$/cm^{-1}	$\Delta I_D/I_G$
Cell 1 Pos. 1	-1.7	-1.1	1.1	3.8	0.00
Cell 1 Pos. 2	0.8	0.1	0.6	-0.7	0.00
Cell 1 Pos. 3	1.0	0.8	-0.4	-1.5	0.01
Cell 1 Pos. 4	0.7	1.0	-0.5	-0.8	-0.01
Cell 2 Pos. 1	-0.1	-0.8	0.0	-1.3	0.01
Cell 2 Pos. 2	1.2	0.3	-0.4	0.2	0.00
Cell 2 Pos. 3	0.7	0.3	0.4	-0.6	0.01
Cell 2 Pos. 4	-0.1	0.1	-0.2	0.6	0.00
Average	0.3 ± 0.9	0.1 ± 0.7	0.1 ± 0.6	0.0 ± 1.7	0.00 ± 0.01

Note that there are unusually large changes for cell 1 position 1. This is probably due to the effect of less wetting of electrode during the first couple of cycles, since position 1 is measured first.

Additional figures regarding the surface reactions during initial cycling

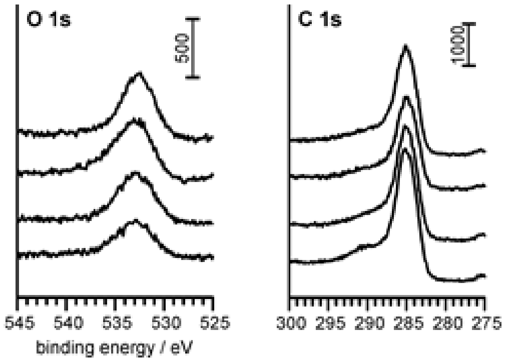

	C	O	I	Na
(i)	96.1	3.9	0	0
(ii)	80.8	9.9	2.5	6.8
(iii)	57.8	24.9	4.9	12.4
(iv)	58.9	23.8	4.5	12.7

Figure S3. XP spectra (O 1s, C 1s) and relative elemental concentration of pristine MSP 20 (i), after immersion in 1M NaI/H_2O (ii) and after cycling with a counter electrode mass ratio of 1:1 (iii) and 1:2 (iv).

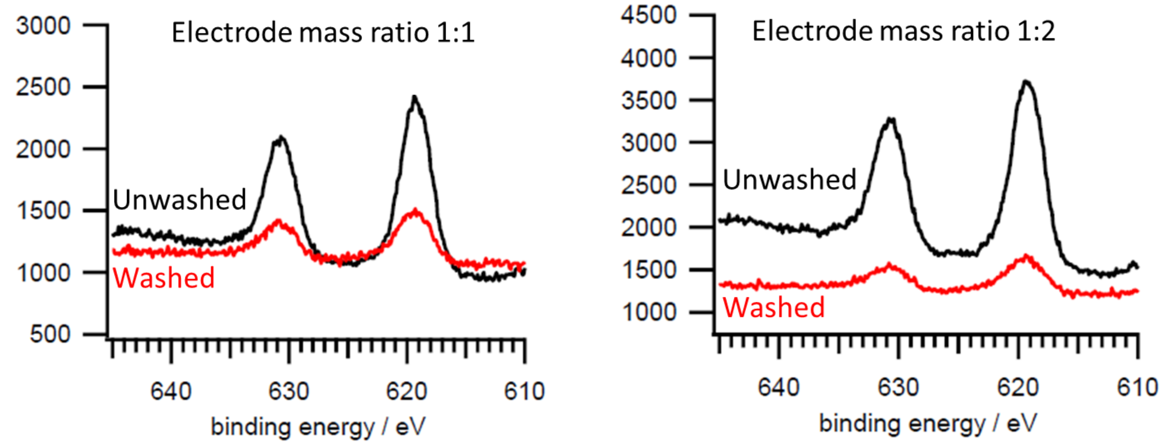

Figure S4. XP spectra of I 3d of MSP 20 electrode after cycling in 1M NaI/H_2O with a counter electrode mass ratio of 1:1 and 1:2.

Additional figures regarding the first contact between carbon and the electrolyte

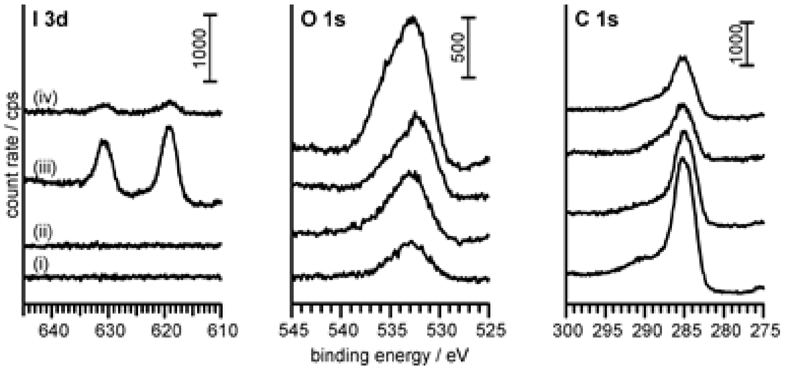

	C	O	I	Na
(i)	96.1	3.9	0	0
(ii)	94.5	5.5	0	0
(iii)	80.8	9.9	2.5	6.8
(iv)	90.3	8.6	0.4	0.7

Figure S5. XP spectra (I 3d, O 1s, C 1s) and relative elemental concentration of pristine MSP 20 (i), after immersion in H_2O (ii), after immersion in 1M NaI/H_2O (iii) and after immersion in 1M NaI/H_2O and subsequent washing in H_2O (iv).

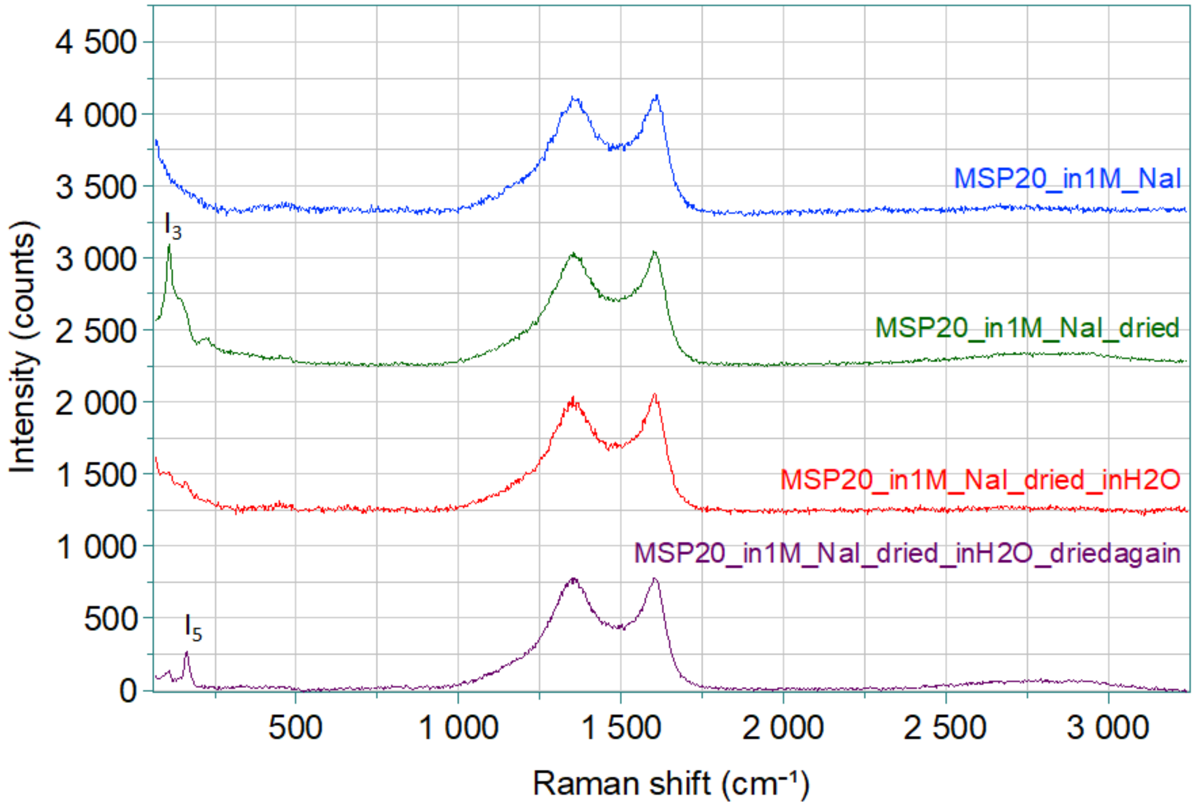

Figure S6. Raman spectra of MSP 20 immersed in 1M NaI/H_2O, dried after immersion (a I_3 band is visible at 110 cm^{-1}), MSP 20 in the water again during washing and dried again after washing (small I_5 band visible at 160 cm^{-1}).

Additional figures methods

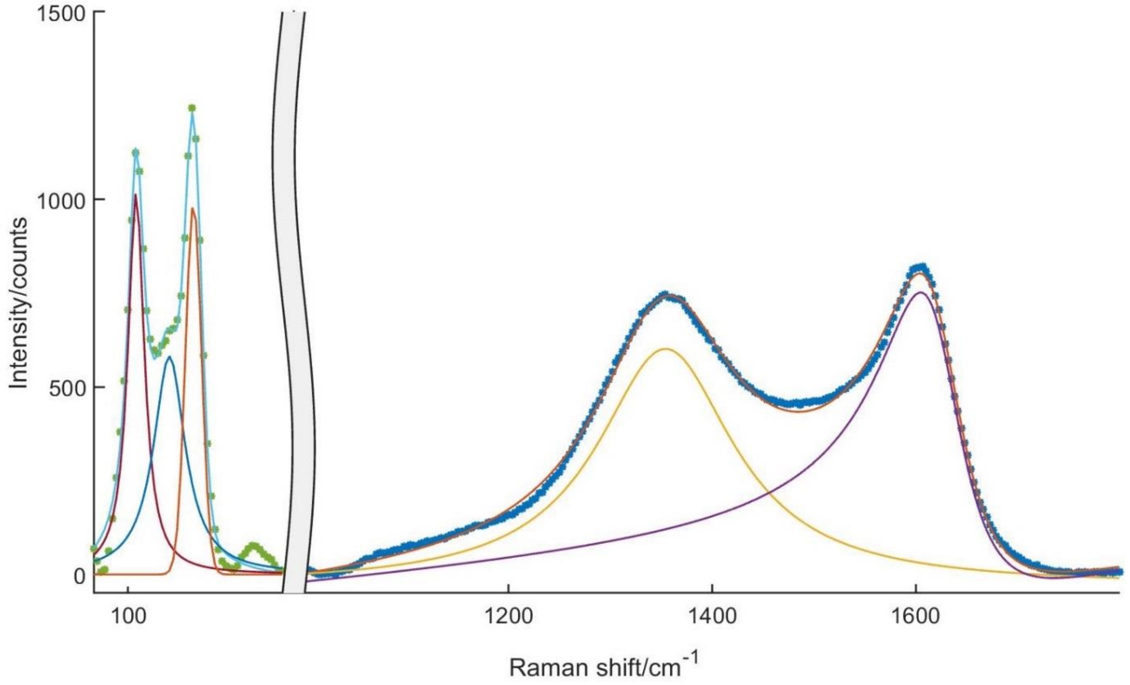

Figure S7. Example of the band deconvolution of the polyiodides (≈100-200 cm^{-1}) region and the G and D bands (1000-1800 cm^{-1}). Note that the overtone of the I_3-band (≈ 230 cm^{-1}) is ignored in the band fit.

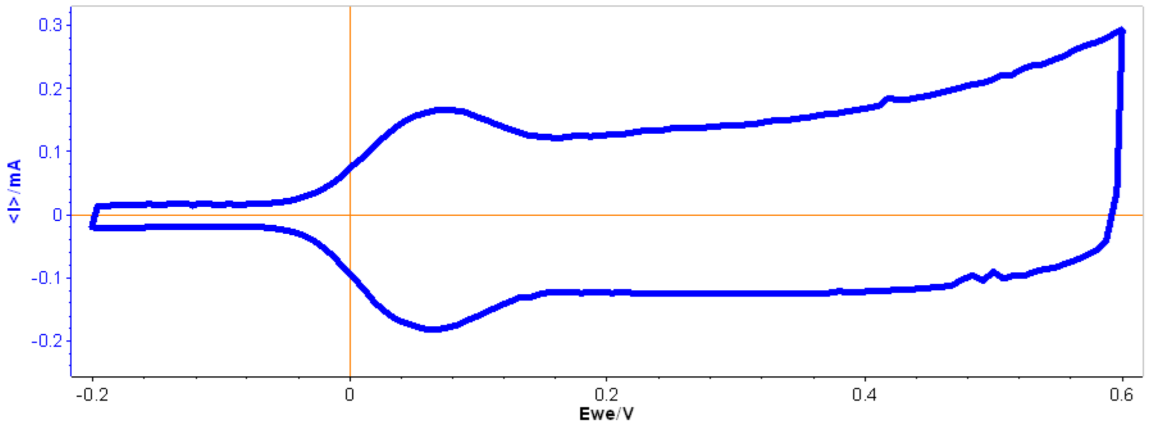

Figure S8. Representative cyclic voltammogram (scan rate = 2 mV s^{-1}) during initial cycling of supercapacitor cell (electrode active carbon mass ratio 1:2) in aqueous NaI. The positive carbon electrode from this cell after 5 cycles was used as sample for XPS analysis.
